# Interpositional substitution of free vas deferens segment autografts in rat: feasibility and potential implications

**DOI:** 10.1186/1471-2490-14-61

**Published:** 2014-08-07

**Authors:** Teoman Cem Kadioglu, Peter D Temple-Smith, Graeme Southwick

**Affiliations:** 1 Department of Urology, Istanbul School of Medicine, University of Istanbul, 34365 Istanbul, Turkey; 2 Department of Obstetrics and Gynaecology, Monash Medical Centre, Melbourne, Australia; 3 Melbourne Institute of Plastic Surgery, Melbourne, Victoria, Australia

**Keywords:** Vas deferens, Autograft, Microsurgery, Fertility, Histology, Spermatozoa

## Abstract

**Background:**

Insufficient vas length for performing a tension-free vasovasostomy is a problem occasionally encountered by microsurgeons. Herein we evaluated utilization of a non-vascularized vas deferens autograft in a rat model.

**Methods:**

Segments of isolated vas deferens, 2.5 cm in length, were used as bilateral autografts in 15 rats. Each autograft was implanted between the two transected ends of vas deferens using end-to-end anastomosis. Fertility, sperm motility, and graft survival was evaluated and compared with the control group.

**Results:**

At the end of the 3 months, 9/15 (60%) rats were able to breed successfully and 24 (80%) vas grafts were patent and viable. Large granulomata developed at the proximal anastomosis sites in 6 (20%) autografts that failed. Unilateral minimal fluid leakage was observed in 6 (20%) of the proximal (testicular end) anastomosis sites in those rats that were able to breed. Histological evaluations demonstrated that graft survival was associated with mild to severe changes in the structure of the vas autograft. On semen analysis 76% of the sperms in the experimental group had forward motility compared to 78% in the control group (p > 0.05).

**Conclusions:**

Vas autograft can successfully be performed in a rat model with ultimate breeding capability.

## Background

The vas deferens is a thick-walled, muscular duct that forms the distal extension of the ductus epididymidis and transmits spermatozoa and seminal fluids from the epididymis and seminal vesicles to the ejaculatory duct [1]. While vas deferens is a target for vasectomy [2], one of the most popular methods of male contraception, it also serves as an important focus in the surgical treatment of obstructive azoospermia [3,4]. Advances in microsurgical techniques during the past 30 years have improved the accuracy and success of vas-to-vas and vasoepididymal anastomoses. New modifications, such as the use of biomaterials/sealants, laser soldering, absorbable and nonabsorbable stents, new intussusception vasoepididymostomy (VE) anastomotic techniques, and robotics, are currently under investigation [5-8].

Due to these technical advancements, recent data indicate that microsurgery appears more cost-effective than percutaneous ‘Testicular Sperm Extraction’ (TESE) and ‘Micro Epididimal Sperm Aspiration’ (MESA) for the treatment of obstructive azoospermia when considering the impact of indirect costs [9]. A tension-free microanastomosis is the key to patency and is often difficult to achieve in some patients because previous surgery has decreased the available length of the vas deferens bilaterally. In a previous study, we developed a technique in rabbits using 1-cm vascularized segments of the vas deferens to bridge selected regions of the epididymis [10]. Based on the results of this study, we reasoned that vas segments could be used in selected clinical situations as autografts. However, due to the anatomical difficulties of retaining a vascular pedicle in human vas segments, an animal model was required to examine the possibility of using free vas autografts in a manner similar to the use of autografts in vascular surgery [11,12]. Although vascular autografts are now in routine clinical use, few attempts have been made to use vas autografts in experimental or clinical situations [1].

The aim of this study is to develop a rat model for vas autograft microsurgery and further examine the survival and function of the free vas autografts. Changes in the interpositional polarity of the vas segment, which were previously believed to interfere with sperm survival and transport in the vas bridge procedure [7], were re-examined using this rat model. Histological evaluation of the vas grafts and comparative fertility assessment of experimental and control groups were performed at three months after surgery as measures of the success of the model.

## Methods

The experiments described in this paper were conducted with the approval of the Monash University Department of Anatomy Ethics Committee and under the national animal in experimentation guidelines on the care and use of animals of the National Health and Medical Research Council of Australia. Also, Animal Research: Reporting In Vivo Experiments (ARRIVE) guidelines for reporting animal studies were adhered.

### Animals

Fifteen mature male Sprague–Dawley rats (90 days old) were used for vas autograft surgery, and ten unoperated males served as controls. These animals were later mated with 30 female rats (two females: one male) to assess fertility (see “Fertility assessment” below).

### Surgical procedures

**Figure 1:**
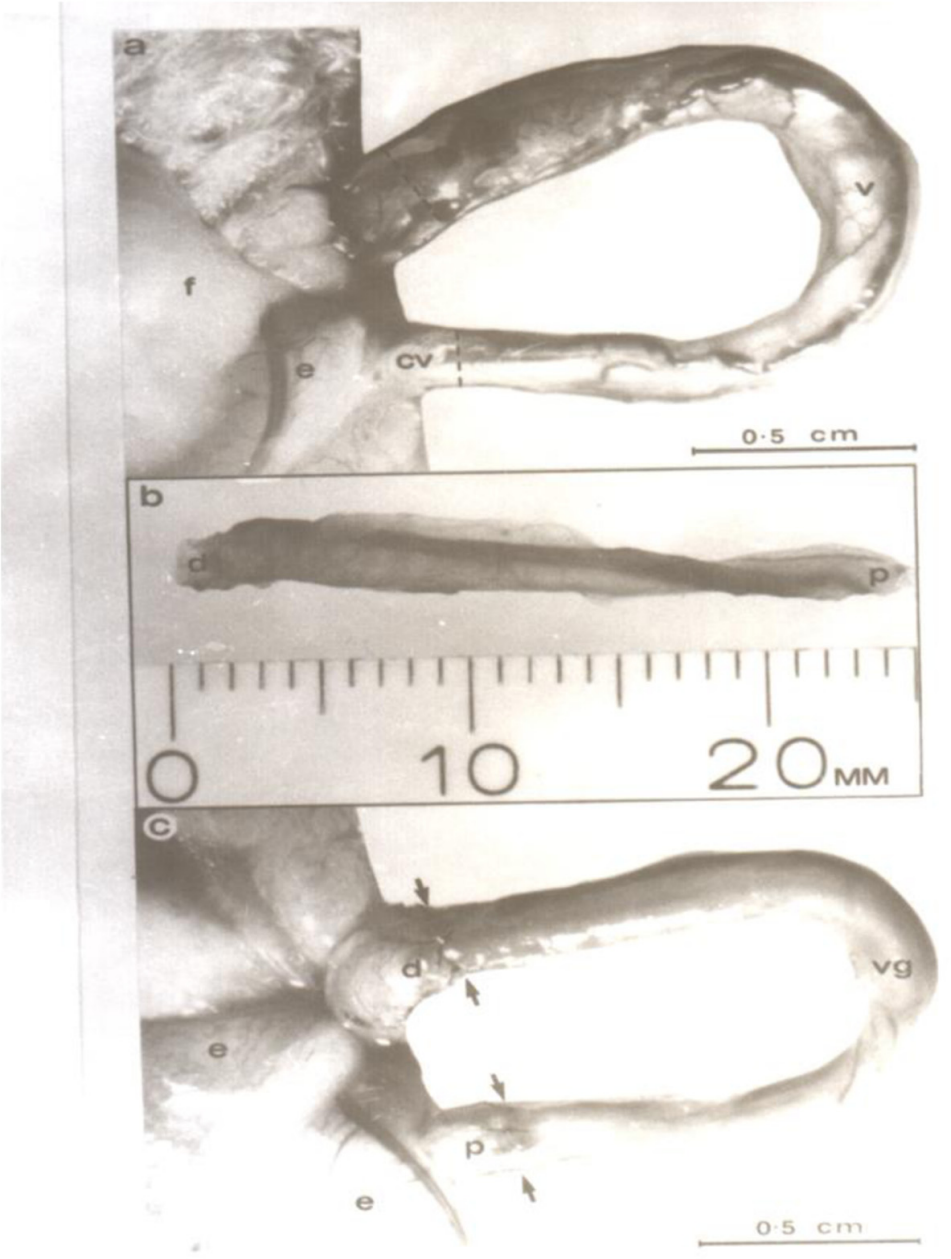
**Vas autograft microsurgical procedure in the rat. (a)** Vas preparation prior to transaction showing a loop of exposed intact straight vas (v) with transaction sites marked (dashed lines). e, epididymis; f, epididymal fat pad; cv, convoluted vas. **(b)** Isolated 2.5 cm vas segment prepared for use as an autograft. Note the increasing diameter from proximal (p) to distal (d), which is associated with the increasing thickness of the smooth muscle layers. **(c)** Vas autograft (vg) in position after microsurgery showing the proximal (p) and distal (d) lines of 10/0 sutures along each anastomosis site.

Following the induction of general anesthesia using sodium pentobarbital (Nembutal 60 mg/ml, intraperitoneal), a 1-cm low, ventral, midline incision was used to expose the abdominal viscera. The testes and spermatic cords were delivered into the surgical field through the inguinal canals using gentle scrotal pressure to expose the vasa deferentia. A 2.5 cm segment of the right vas was gently freed from the surrounding tissues from the junction of the convoluted and straight vas distally (Figure 1a). Major vessels at the proximal and distal points of transection of the vas were cauterized with microforceps using bipolar cautery, and the vas, along with its associated neurovascular supply, was then severed with microscissors. The same procedure was applied to the distal end of the dissected portion of the vas to produce a 2.5-cm isolated vas segment for use as an autograft (Figure 1b). Care was taken to preserve the integrity of the testicular blood supply. Prior to excising the autograft, the deferential vessels were cauterized immediately proximal and distal to the proposed graft site. The graft segment was detached from the vas using microscissors and placed in Ringer’s solution at room temperature. The ischemic time in cold Ringer’s solution was less than 20 minutes. A similar segment from the left vas deferens was then excised for cross-grafting. The free portion of the right vas was then interposed as an autograft between the transected ends of the left vas. The proximal and distal anastomoses were performed under 9-25× magnification using a single layer of five full-thickness 10–0 monofilament nylon sutures (Figure 1c). These sutures were placed equidistant around the vas to ensure an exact approximation of the lumen at each end of the autograft and the two severed ends of the vas. The left vas and its associated testis and epididymis were then returned to the scrotum. The same procedure was performed on the right using the contralateral vas segment and the midline ventral incision was closed in a two-layer fashion using 6–0 nylon sutures for the body wall and 6–0 silk sutures for the skin.

The polarity of the vas autografts was reversed in a sub-group of five rats, such that the proximal end of the autograft was anastomosed to the distal end of the *in situ* vas, and the distal end of the graft was joined to the proximal *in situ* vas.

### Fertility assessment

On the 20^th^ postoperative day, two mature female rats were placed in the cage of each male rat. The abdomens of these females were gently palpated daily for evidence of pregnancy. At the first sign of pregnancy in either female, both female rats were isolated from their male consort. The first of the two female rats showing evidence of pregnancy by palpation of the abdomen was followed carefully throughout gestation. At the time of littering, the date and the number of offspring were noted. The date of conception was then calculated based on the 21-day gestational period for rats.

Control animals were exposed to females at the same time when the experimental rats were evaluated for establishment of pregnancies.

Three months after cross-grafting, those males that were unsuccessful in establishing a pregnancy were separated from their female consorts for autograft evaluation. A mature, male, unoperated, control rat was placed with the remaining nongravid females to eliminate the possibility of female infertility as a confounding factor.

### Sperm motility studies

Sperm motility was assessed in all of the experimental animals after fertility assessments were completed. The right vas was cut 3 mm distal to the distal suture line, and spermatozoa were retrieved using a standard microaspiration technique [13]. The percentage of motile spermatozoa in each sample and their motility indices were calculated using the same routine visual analysis procedures recommended for human semen analysis. Sperm samples were also microaspirated from the convoluted vas at a level 3 mm proximal to the proximal suture line and analyzed as described above. Similar sample collection procedures and motility analyses were used to assess the motility of spermatozoa from the contralateral side. Similar sperm motility assessments were also performed on the ten unoperated control rats. Within-animal data were compared using Student’s t-test, and sperm motility data from the same anatomical levels in experimental and control rats were compared using a one-way ANOVA (Minitab Statistical Software Inc., Pennsylvania, USA).

### Autograft evaluation

**Figure 2:**
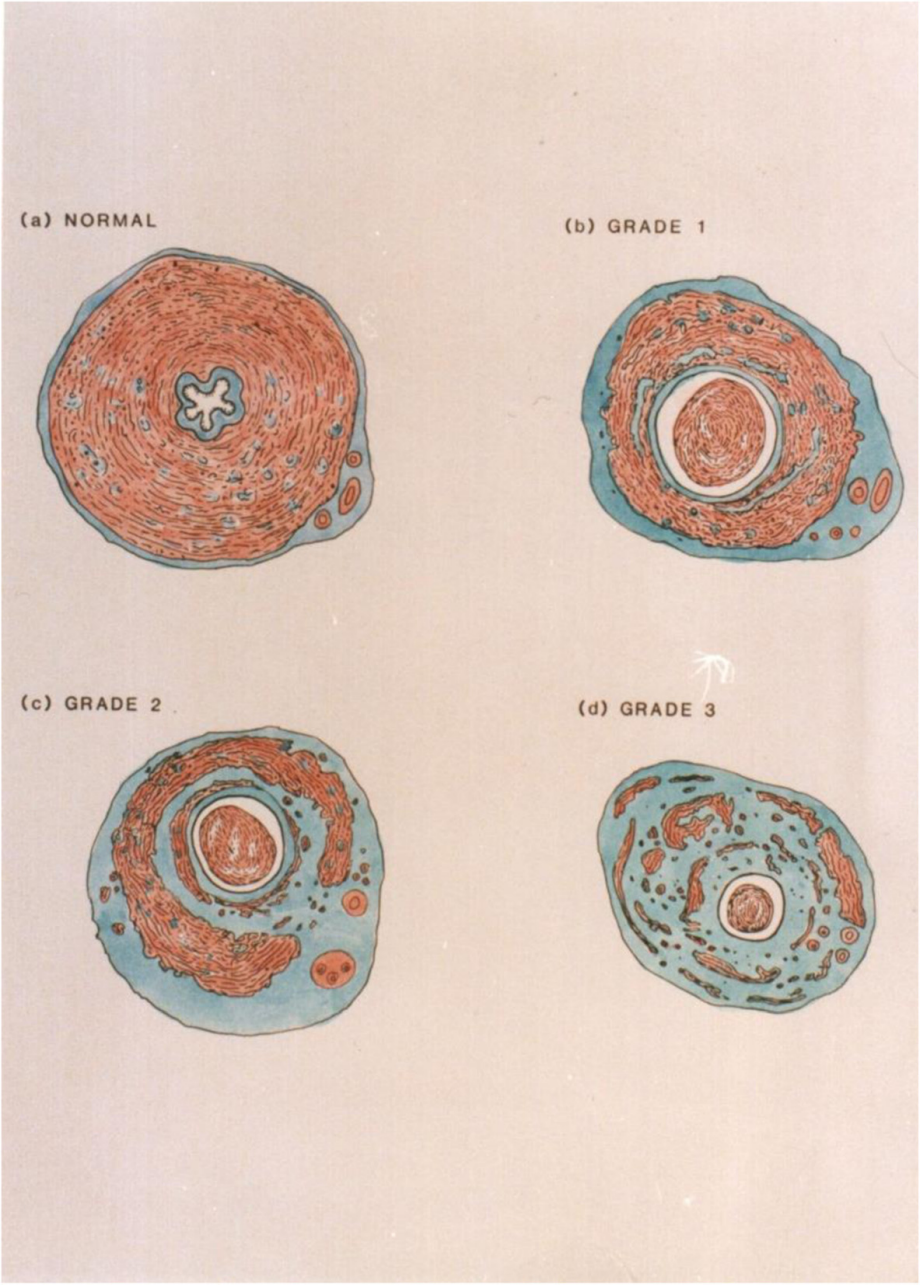
**Grading system. (a)** Grade 0: Control vas graft; normal histology. **(b)** Grade 1: Loss of stellate lumen but maintenance of periluminal smooth muscle continuity. **(c)** Grade 2: Reduction in mass and progressive fibrosis of the longitudinal and circular smooth muscle causing discontinuity of muscle layers. **(d)** Grade 3: Smooth muscle layers are largely replaced by fibrous tissue surrounded by a revascularizing, thickened adventitia.

After 90 days, fertility assessments were completed and each rat was sacrificed. The viability of the vas grafts evidenced by the presence of spermatozoa inside each autograft and in the adjacent vas above and below the graft were examined under low power magnification (9×) in all 15 experimental animals. The patency of the autografts was evaluated under 9× magnification. The presence of a continuous white column of seminal fluid throughout the autograft and into the distal (abdominal) vas was indicative of graft patency. The presence of anastomotic leakage or granuloma was also documented. The autograft, including the proximal and distal anastomotic sites, was excised, preserved in Bouin’s solution, and prepared for light microscopy. Longitudinal sections were cut through the anastomoses for further study of the operative sites. Four-micron-thick transverse sections were cut through the center of each autograft and from regions adjacent to the proximal and distal anastomosis sites to examine the vas autografts for any obvious histological changes. Sections stained with hematoxylin and eosin and Masson’s trichrome were prepared from each paraffin block. Similar histological observations were made on vas samples obtained from similar regions in each of the ten unoperated control rats.A grading system was devised to objectively assess the microscopic changes in autograft histology. The extent of smooth muscle degeneration was the basis for this system (see Figure 2 for a drawing of the vas grading system (0–3) with an appropriate description in the legend).

## Results

### Fertility assessment

Nine of the 15 (60%) rats established a pregnancy with at least one of the two females introduced into their cages following the twenty-day postoperative period of celibacy. Three pregnancies were achieved in rats with reversed polarity grafts and six in rats with normal polarity grafts. The fact that the same percentage (60%) was achieved in both groups shows consistency. The fertility of all twelve (six pairs) of the female rats that failed to establish pregnancy during cohabitation with postoperative males was confirmed through breeding with control males.

### Graft survival and patency of the anastomosis

**Figure 3:**
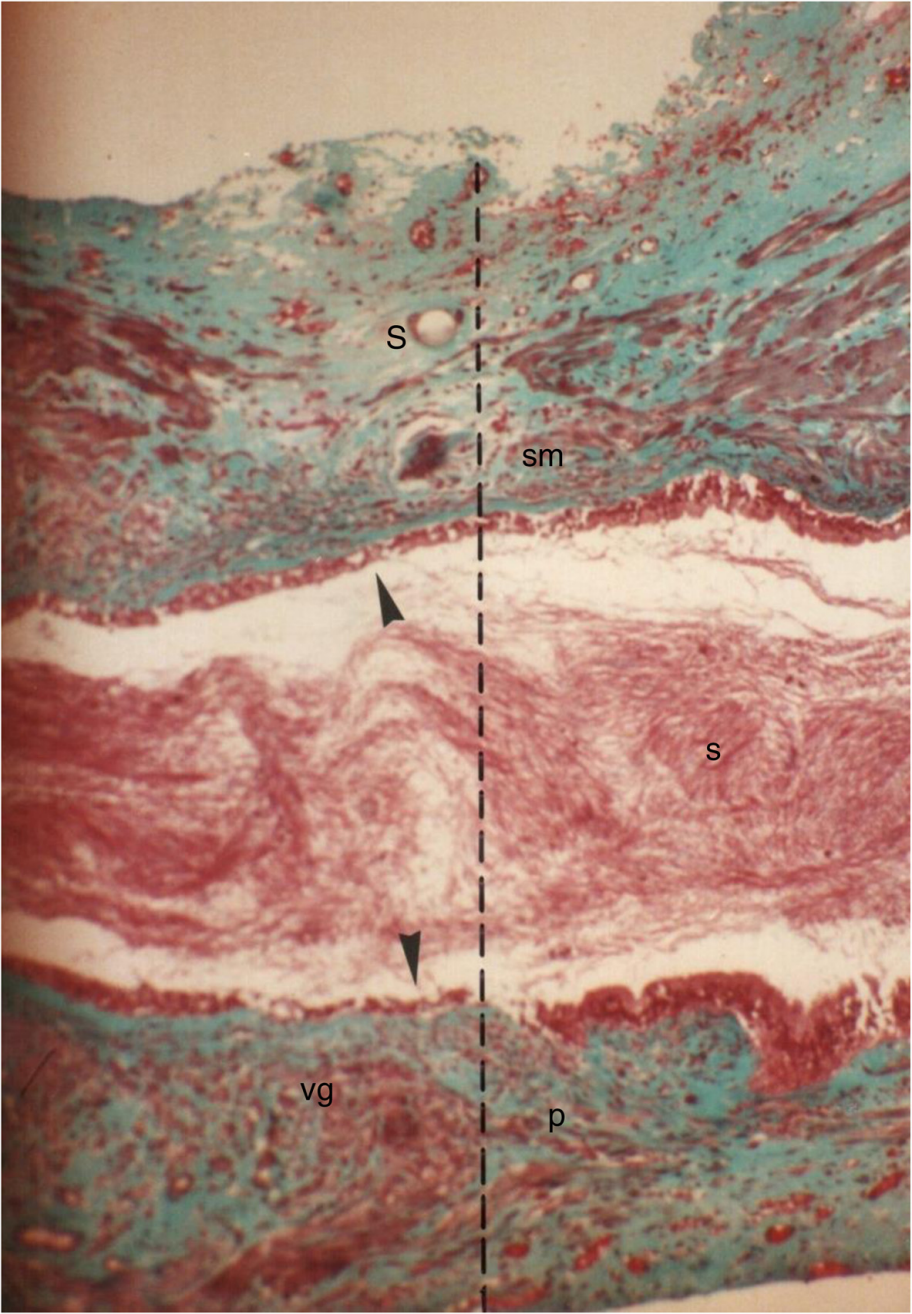
**Longitudinal section through the proximal suture line of a vas autograft showing the patency of the anastomosis.** The spermatozoa (s) filling the lumen, the suture (S), and the interrupted smooth muscle (sm) layers along the anastomosis site (dashed lines) are shown. Proximal vas (p), vas autograft (vg). Note the reduced length of the stereocilia (arrows) in the vas autograft 135x.

**Figure 4:**
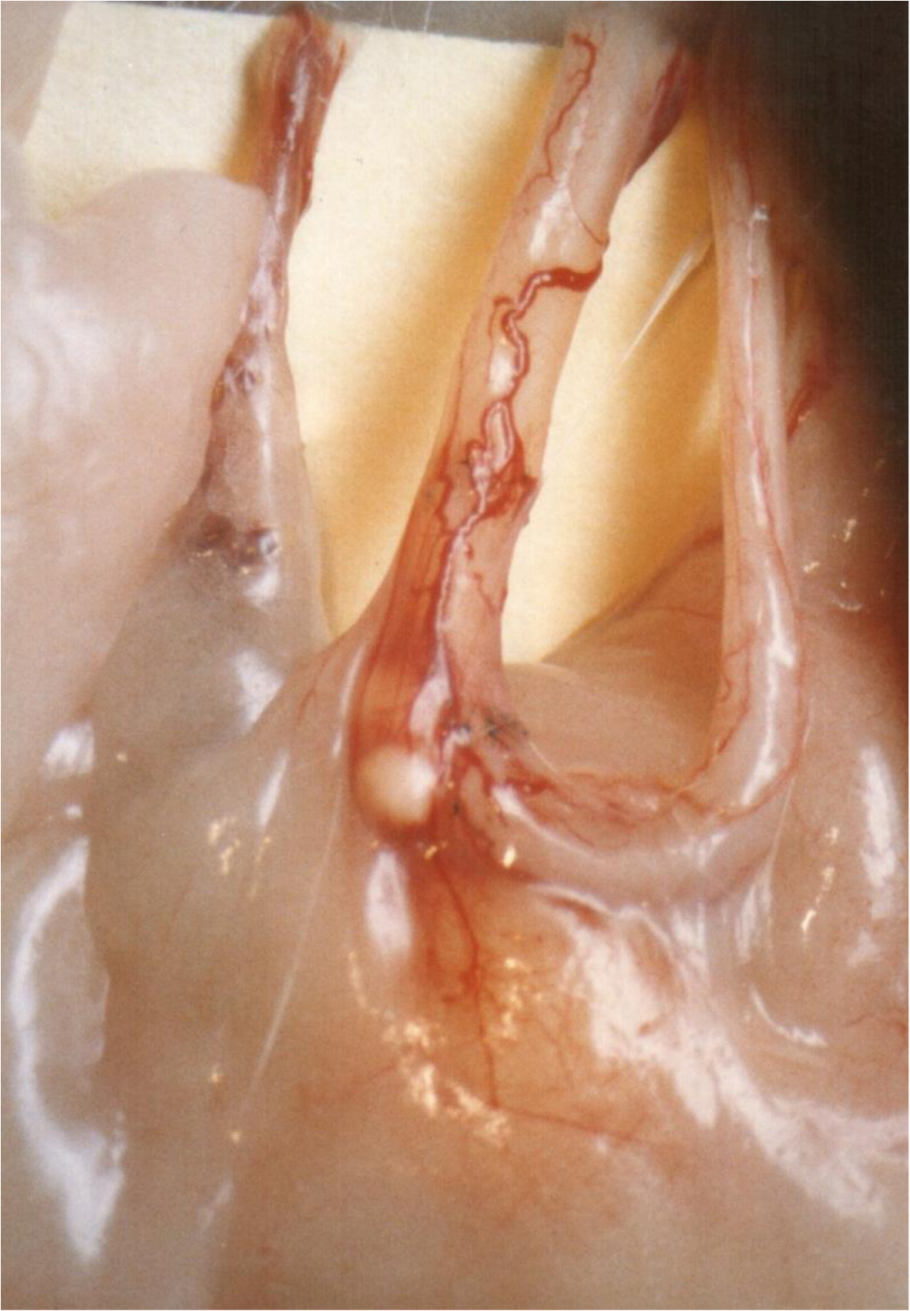
Minimal leakage at the proximal anastomosis site.

**Figure 5:**
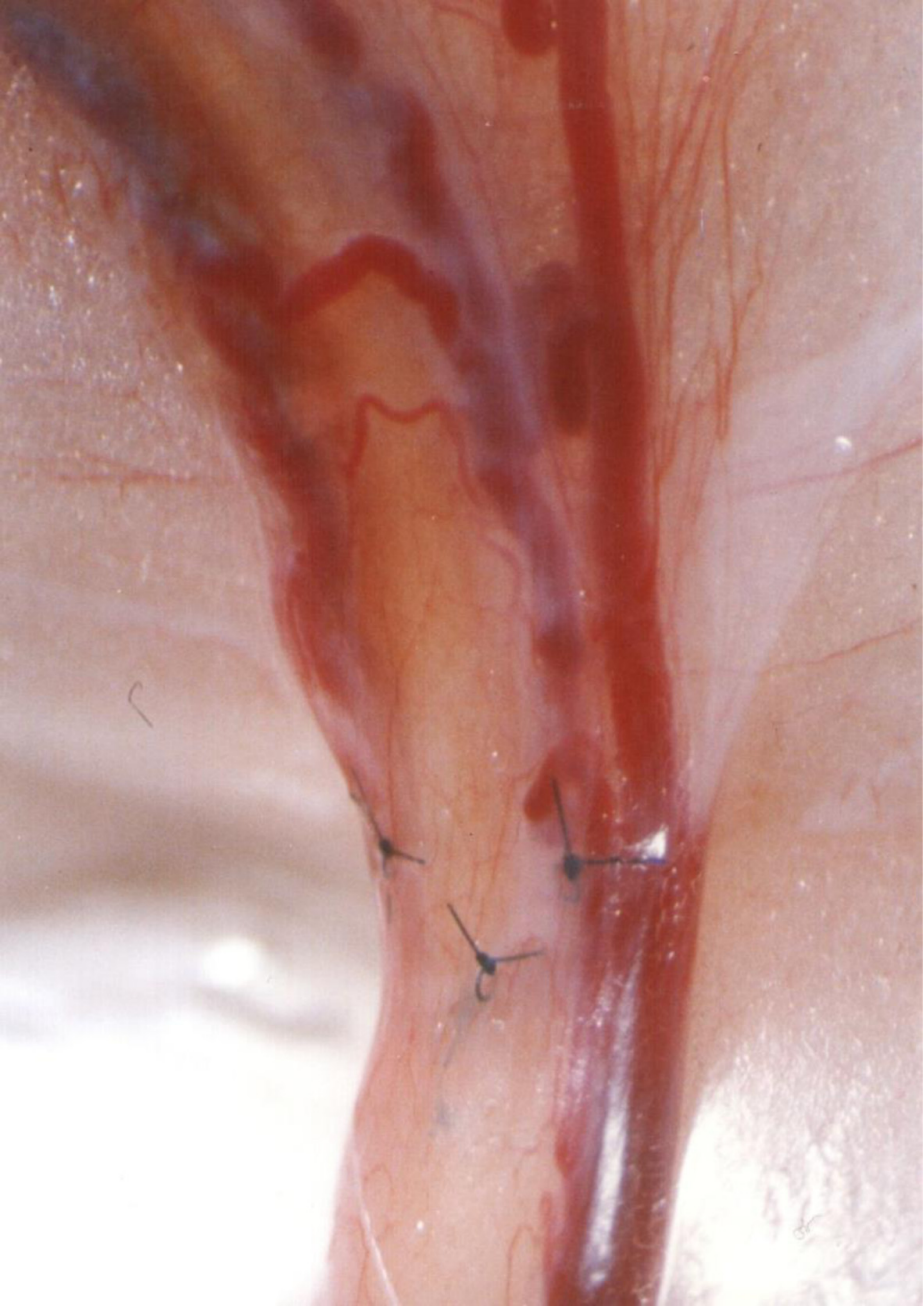
Prominent neovascularity.

Graft patency, tissue quality, and neovascularity were examined. Of the 30 vasautografts performed, 24 (80%) were patent and viable (Figure 3). Minimal unilateral fluid leakage was observed at six (20%) of the proximal anastomosis sites in those rats that had established pregnancies. No adverse effect of this leakage was observed on either graft patency or survival (Figure 4). Prominent neovascularity (Figure 5) was observed in all surviving autografts at 90 days. Large unilateral granulomata developed at the proximal anastomosis sites of the six (20%) autografts that ultimately failed. Histological degeneration was more profound in the vas graft segments immediately adjacent to these granulomata. The polarity of the autograft appeared to be inconsequential because 8 (80%) of reversed polarity grafts survived and the grafts that did not survive also had large unilateral granuloma at their proximal anastomosis sites.

### Histological examinations

#### Grafts

**Figure 6:**
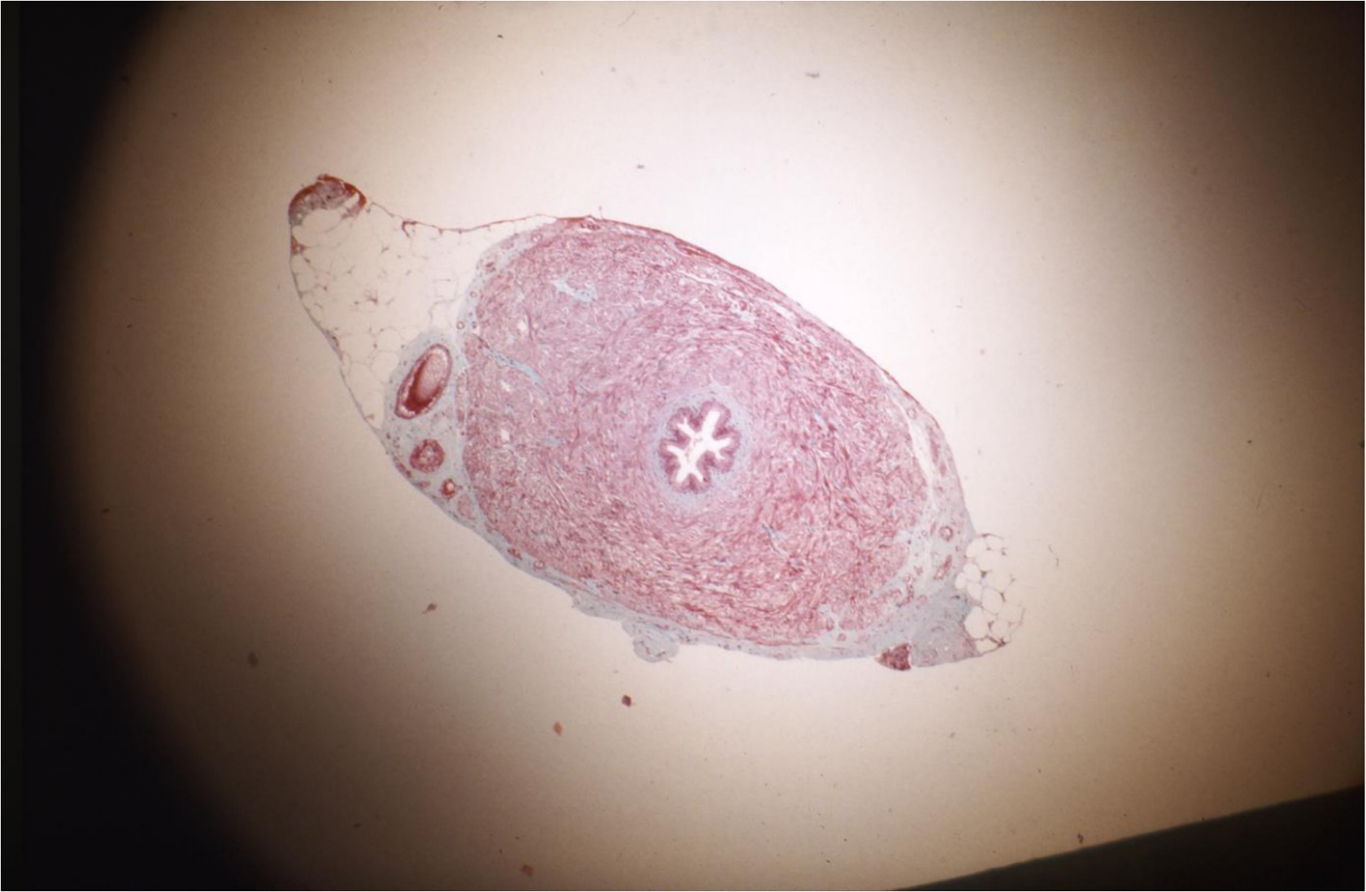
Normal vas deferens.

**Figure 7:**
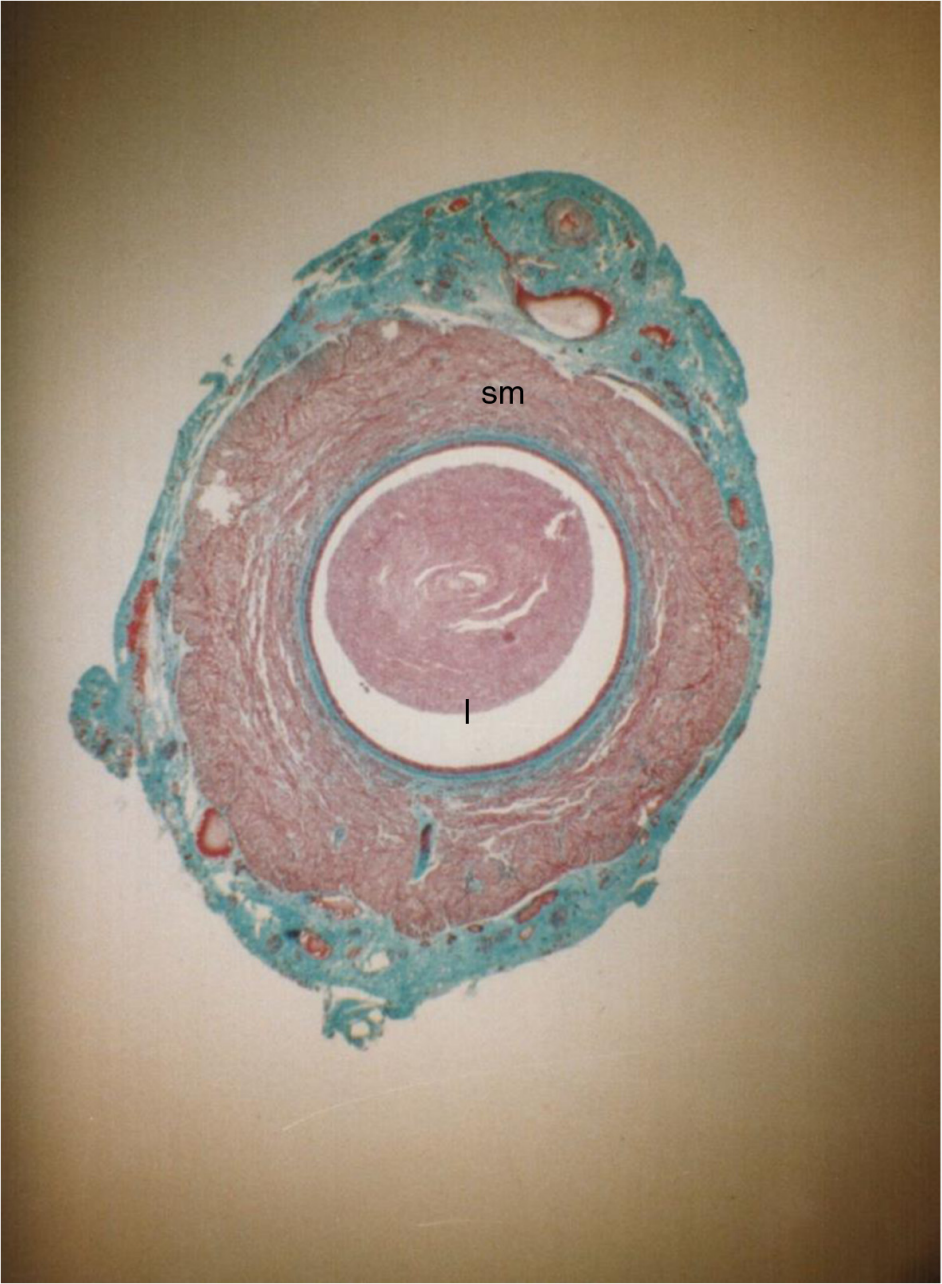
**Transverse section through a vas autograft 1 month after surgery showing minimal changes.** The slightly dilated lumen (l) has partially lost its stellate shape and contains few spermatozoa, and the smooth muscle (sm) layers have remained intact with thickened, revascularized adventitia (arrows). Note the presence of stereocilia on the epithelial cells as in controls 55x.

**Figure 8:**
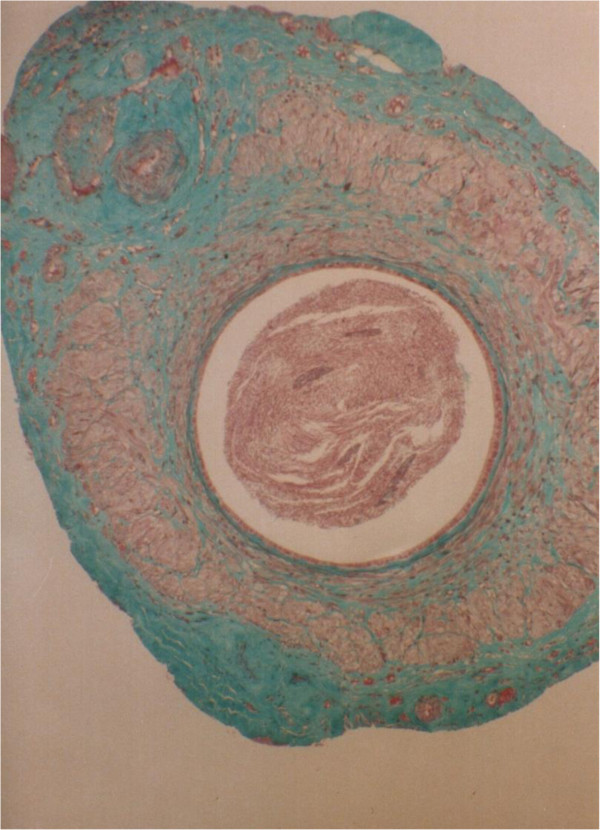
**Transverse section through a vas autograft 1 month after surgery showing mild degenerative changes.** The enlarged, rounded lumen (l) exhibits an increased presence of spermatozoa (s), and disrupted smooth muscle (sm) layers are partially replaced by fibrous tissue (f) surrounded by a revascularizing, thickened adventitia (arrows). Note the reduced epithelial height (arrow heads) and large perivasal pad of adipose tissue (a) 55x.

**Figure 9:**
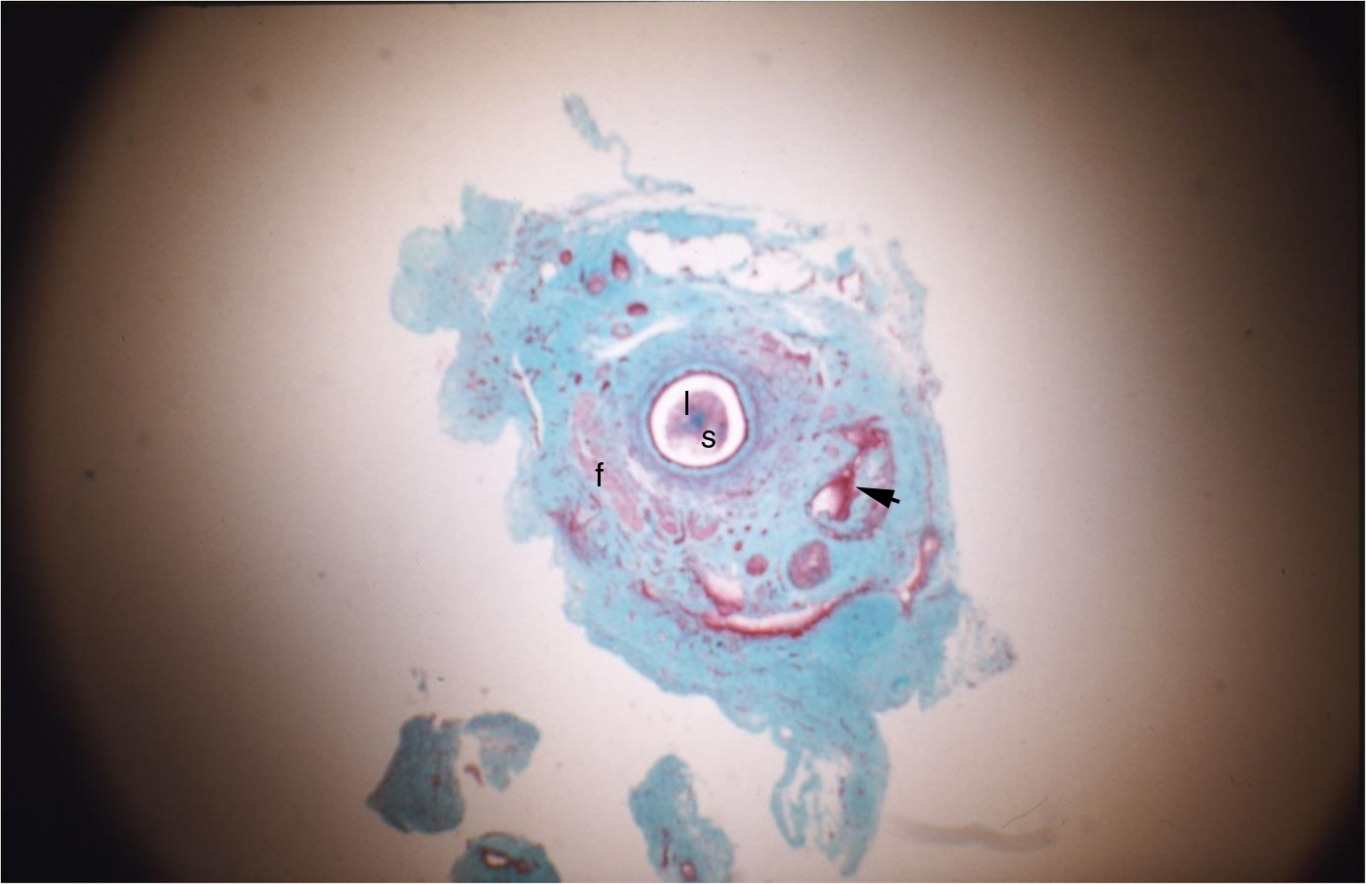
**Transverse section through a vas autograft 1 month after surgery showing severe degenerative changes.** The rounded lumen (l) exhibits an increased presence of spermatozoa (s), and smooth muscle (arrow heads) layers are largely replaced by fibrous tissue (f) surrounded by a revascularizing, thickened adventitia (arrows) 55x.

All grafts showed some structural changes when compared with the controls (Figure 6). Light microscopic examination of tissue sections from the mid-portions of the viable autografts showed that they had all undergone some degeneration of the smooth muscle layers. The normal stellate configuration of the vas lumen was lost in all of the grafts. The lumina had become circular and smooth in appearance.Two (6.6%) of the autografts demonstrated only grade 1 changes (i.e., loss of stellate lumen but maintenance of periluminal smooth muscle continuity) (Figure 7). Sixteen (53.3%) of the grafts showed grade 2 changes (Figure 8), and 12 (40%) showed grade 3 changes (Figure 9). In addition to fibrosis of the smooth muscle layers, we observed decreases in the luminal epithelial cell height and stereocilial length in all (100%) of autografts. Neovascularity was continuous with the adventitial blood supply of the proximal and distal native vas segments. Increased branching of the superficial vessels was observed as they traversed the anastomosis and supplied the autograft.

#### Observations of the spermatozoa

The presence of sperm distal to the patent grafts was confirmed by microaspiration. Motile spermatozoa were recovered from vas segments adjacent to the graft. No significant difference was observed in the percentage of motile spermatozoa, in the sperm motility index between samples collected from sites proximal and distal to the graft (Table 1), or between normal and reversed polarity patent grafts. Sperm motility parameters derived from the experimental rats did not differ significantly from those of sperm collected from comparable sites in the control rats (Table 1).

**Table 1:** Comparison of the percentage of motile spermatozoa and motility indices in sperm samples microaspirated from above and below the vas autografts (n = 15) and from identical vas sites in control animals (n = 5)

	**Motility assessment**		
	**Control (n = 5)**	**Experimental (n = 15)**	**p**
Proximal collection site			
% motile	59.1 + 24.6	51.1 + 12.3	0.34
Motility index	99.7 + 43.4	91.7 + 27.2	0.62
Distal collection site			
% motile	64.1 + 23.6	61.2 + 17.4	0.77
Motility index	109.8 + 47.1	102.2 + 28.9	0.66

No comparisons between the data using either Student’s t-test (within animal data pair analysis) or one-way ANOVA (between animal data comparison) were found to be significantly different.

## Discussion

The high graft survival and patency (80%) described in this study shows that vas grafts can be successfully auto-transplanted in rats via microsurgical techniques. The pregnancy data and post-mortem graft examination further substantiate the functional success of free vas autografts. A similar study was performed by Carringeret and associates using smaller vas segments or avascular grafts [14]. Although they did not evaluate pregnancy or the quality of spermatozoa, they did show that neovascularization of the transplanted vas grafts facilitated graft survival and patency.

Although extensive studies have demonstrated the superiority of a two-layer or modified one-layer microsurgical anastomosis in humans [6-8], only a simple, single layer anastomosis was technically feasible in the very thin, proximal rat vas. One of the major difficulties in using the rat model for developing a vas autograft is the predilection of the rat to develop granulomas [4,5]. The granulomata that were found adjacent to the proximal suture lines may have been caused by a pressure gradient forming in front of the hypoactive or inactive vas segment. It is noteworthy that the granulomata were found at the proximal anastomosis of each of the obstructed grafts. In men, granulomata formation at anastomotic sites plays a role in the postoperative results of vasectomy reversal [10]. Due to both the ability to perform a more watertight anastomosis and the lower incidence of granulomata, the use of the vas autografts in men would be less likely to suffer from obstruction. The low number of granulomata (3/30) found adjacent to the proximal anastomosis sites might have been due to the single layer anastomosis in rat vas autografts.

In contrast to the common development of convolutions in straight vas segments used in vasoepididymal anastomoses and vas-bridge procedures in rabbits [10], no convolutions developed in any of the rat vas autografts at one month after surgery.

Despite the progressive nature of fibrotic changes in the smooth muscle layer of the grafts, the survival, revascularization, and early patency of these free autografts remain encouraging. The most obvious of these changes included the reduction in mass and progressive fibrosis of the layers of the longitudinal and circular smooth muscle and the associated changes in the luminal shape. In fact, there was an apparent relationship between the luminal shape and degeneration of the smooth muscle layers. In the grafts that were most severely affected, the lumen was round and packed with spermatozoa; in contrast, those in which the smooth muscle layers remained essentially unaffected exhibited a luminal shape more similar to the characteristic empty, stellate lumen of control vasa. This observation suggests that the change in luminal shape is caused by the loss of smooth muscle tone in the vas autografts. Two obvious factors may have led either separately or synergistically to the degenerative changes: 1) the temporary loss of the vascular supply and/or 2) the transection of nerves to the vas segment used as autografts. Similar degenerative changes have been reported in the smooth muscle layer of vascular grafts [15]. The structural changes occurring in all of the grafts suggested that transection of the nerve and blood supply altered the morphology of the vas without affecting its essential functions of sperm transport and maintenance of sperm viability. It is presumed that either sufficient smooth muscle activity to enable some transport was retained in the autograft segment or that backpressure in the epididymis was sufficient to provide flow of luminal contents through the enlarged autograft lumen. The similarities in sperm motility of the samples taken above and below the graft, and the direct comparison of these with samples from equivalent regions in the control vasa, indicate that the histological changes observed in the epithelium of the autografts had no significant effect on sperm motility, sperm viability, or the autograft luminal environment.

None of the rats in this study were mated until at least twenty days postoperatively to ensure that no viable sperm remained in the distal vas deferens at the time of conception. Kuwahara and Frick reported that more than 90% of the sperm remaining in the vas were decapitated, with motility falling to almost zero at six days following vas ligation [16]. Although sperm can survive for long periods in the epididymis, this study demonstrated that the vas was not conducive to long-term sperm survival.

Our previous work with pedicled vas bridges in rabbits suggested that the polarity of the graft might affect sperm survival and subsequent pregnancy as the sperm transit the graft. Unlike the pedicled bridges, which technically require a polarity change to complete the anastomoses, the polarity of the free vas autografts could be changed easily. In the present study, the graft survival, incidence of granulomata, and pregnancy rate were unaffected by graft polarity.

The clinical applications for successful vas autografts in men are potentially far-reaching. Insufficient vas length for performing tension-free vasovasostomy is a problem occasionally encountered by reproductive microsurgeons. Excessive scarring secondary to previous operative procedures and aggressive removal or extensive electrocautery of large vas segments during vasectomy are the most common causes of insufficient length for vasovasostomy. For patients presenting with the absence or atrophy of one testicle and insufficient vas length on the contralateral side, a vas autograft would be an ideal surgical option. Even in those men with two normal testes, using one vas as a free graft is attractive when both sides have long segments of scarred or absent vas deferens.

## Conclusions

Translation of the autograft procedure from the rat model to a clinical procedure in men may improve the success of vasal microsurgery in cases where insufficient vas is available bilaterally to provide for tension-free vas anastomoses.

## Competing interests

The authors declare that they have no competing interests.

## Authors’ contributions

TCK performed the operations, analysed and interpreted the data as well as preparing the manuscript. PDTS set up the scientific base for the concept for this study as well as previous similar animal experiments that led the path to this study and designed the study. GS tutored the microsurgical skills and perfected the microsurgical technique. All authors read and approved the final manuscript.

## Pre-publication history

The pre-publication history for this paper can be accessed here:

http://www.biomedcentral.com/1471-2490/14/61/prepub
